# Multi-Annual Fluctuations in Reconstructed Historical Time-Series of a European Lobster (*Homarus gammarus*) Population Disappear at Increased Exploitation Levels

**DOI:** 10.1371/journal.pone.0058160

**Published:** 2013-04-03

**Authors:** Andreas Sundelöf, Valerio Bartolino, Mats Ulmestrand, Massimiliano Cardinale

**Affiliations:** 1 Institute of Marine Research, Department of Aquatic Resources, Swedish University of Agricultural Sciences, Lysekil, Sweden; 2 Department of Earth Sciences, Gothenburg University, Gothenburg, Sweden; University of Bologna, Italy

## Abstract

Through the history of ecology, fluctuations of populations have been a dominating topic, and endogenous causes of fluctuations and oscillations have been recognized and studied for more than 80 years. Here we analyzed an historical dataset, covering more than 130 years, of European lobster (*Homarus gammarus*) catches. The data shows periodic fluctuations, which are first dampened and then disappear over time. The disappearance of the periodicity coincided with a substantial increase in fishing effort and the oscillations have not reappeared in the time series. The shifting baseline syndrome has changed our perception of not only the status of the stock, but also the regulating pressures. We describe the transition of a naturally regulated lobster population into a heavily exploited fisheries controlled stock. This is shown by the incorporation of environmental and endogenous processes in generalized additive models, autocorrelation functions and periodicity analyses of time-series.

## Introduction

Population fluctuations and their causes have been debated for almost a century (e.g. [1], [2], [3]) and are still a central topic for contemporary ecology (e.g., [4]). Causes of population fluctuations and their variability have been hypothesized to derive essentially from 1) environmental forcing (including anthropogenic forcing), 2) species interactions and 3) internal processes such as density dependent regulation of recruitment or survival. Several recent studies have shown strong support for the first two hypotheses but little support for the third [4], [5].

From theoretical models internal processes are known to regulate stock size and cause fluctuations in abundance by, for example, well known over-compensatory recruitment regulations [3]. The theoretical underpinning of many population models suggest that high growth rates cause density dependent fluctuations [2], [6]. We will here make the distinction between fluctuations, that have a stochastic or irregular component, and oscillations that are inherently periodic. The periodicity of oscillations is further sensitive to population structure [7]. In fisheries models such oscillations are maintained at a moderate-low fishing mortality and diminished at high fishing mortality [6]. Thus, harvesting is generally damping oscillations but promoting fluctuations of populations with high growth rates.

On the other hand, fishery has been shown to increase the variability of stock abundance through the truncation of the population age structure, making populations less resilient to environmental variability [8]. Fishery also affects life history traits and may cause an earlier age of maturation [5], [9]. Changes in maturation may be plastic [10] or irreversible [11] and increase variability in recruitment and stock sizes by forcing populations to more closely trace environmental variability [4], [5], [12]. Lately, several studies have shown that endogenous processes are weak in promoting fluctuations compared to harvesting [4], [5], but they all referred to heavily exploited populations. Thus, the question becomes: how are population growth and stock size regulated at low exploitation rates?

**Figure 1:**
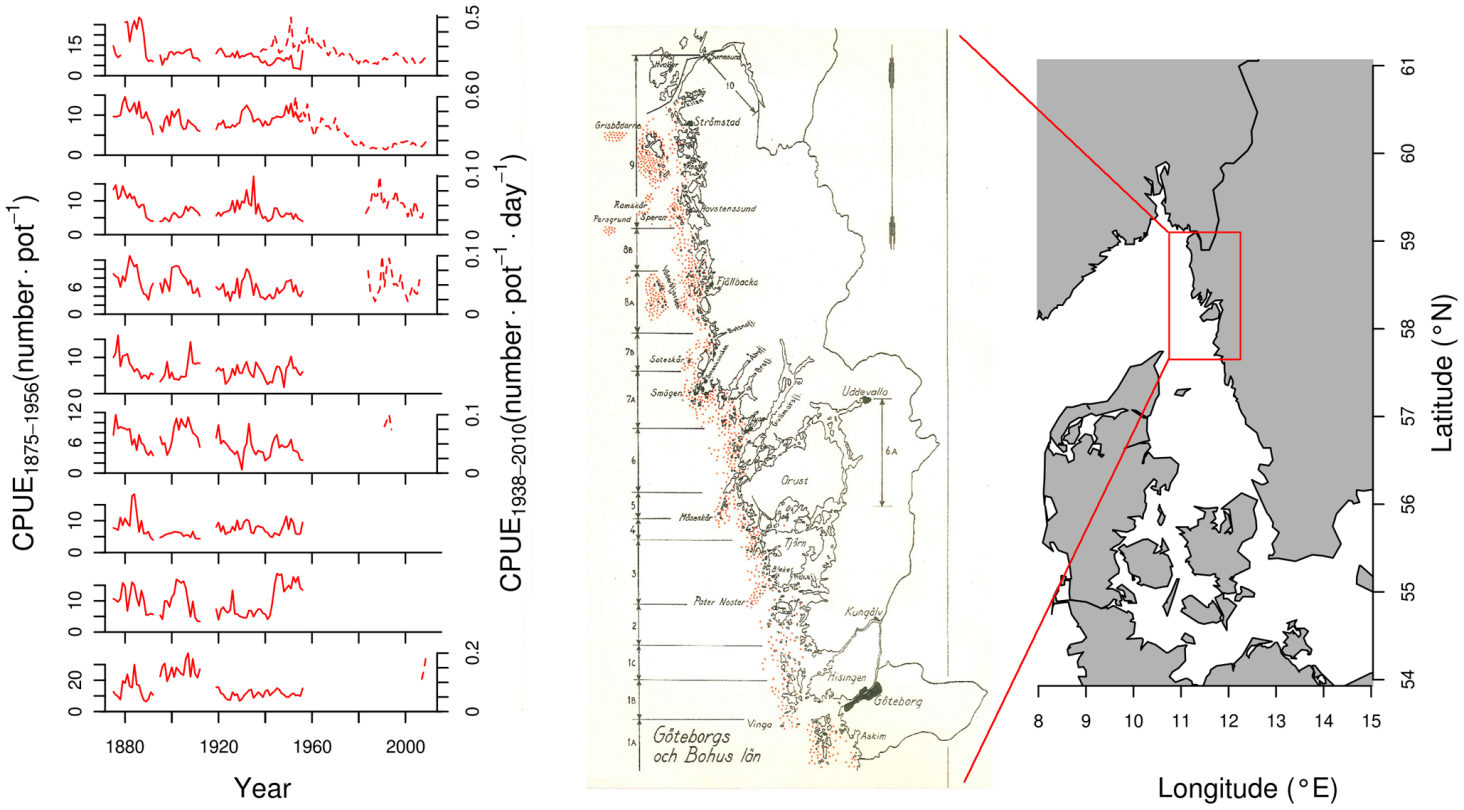
The time series of lobster CPUE were obtained from the West coast of Sweden and used in the analysis. Data from the two sources are presented by area (bold lines are SREAS data and dashed lines are the VCD data) (note the different axes). The different areas are indicated on the hand drawn map from 1942 (Axelsson 1944).

In this paper we present a unique time series of European lobster (*Homarus gammarus*) catches from the Skagerrak, eastern North Sea, developing from a lightly exploited to an overexploited phase (Figure 1). European lobster has been fished for centuries and harvesting for export was introduced in Sweden already during the 17^th^ century [13]. Lobster landings in Scandinavia have since then gone through major fluctuations but are today much smaller compared to historical records [13]. Although landings are uncertain before 1875, nowadays estimates are in the order of one third of the amount landed during the early 1930 s and landings have been even larger in Scandinavian waters in the 1800s. For example, in 1865, two million lobster individuals were exported live from Norway to England [14], amounting to approximately 1000 metric tonnes. This is about 20 fold the catch registered today in Norway.

In Sweden, historical statistics on catch per unit effort (CPUE) from 1875–2010 show a slight decline during the first 80 years followed by an abrupt decrease of CPUE during 1950–75 that set the stock at the lowest observed level. After 1950 s, we do not have data on total effort and total landings but information on stock trends is maintained by fishermen providing detailed data on catch per unit of effort (CPUE, expressed in number of lobster caught per pot per fishing day). This constitutes a complete data set, which provides detailed information on the stock development from the 1870 s to modern times. The stock shows some intriguing fluctuations. The first half of the time series is structured by a pronounced oscillation, the second half by a major decline. Here we analyze spatial and temporal patterns of this time-series to quantify the oscillation, the transformation of the oscillation concerning periodicity and amplitude and subsequent disappearance from the catch data.

The management regime of European lobster (*Homarus gammarus*) in Sweden has been largely unchanged during the analyzed time series. The fishery was first regulated in 1830. A seasonal closure was introduced during 1^st^ July and 15^th^ September almost 100 years after it was first proposed. In 1879 the minimum landing size was set to 21 cm TL. After the great decline during 1950–70 the MLS was adjusted to 22 cm TL in 1973. In 1985 a female moratorium was established and in 1994 a further adjustment of the MLS was done to 80 mm CL (23 cm TL). In 2003 a general ban on fyke nets was put on the lobster fishery. There is no quota or bag limit to regulate daily or yearly catches. Effort is regulated only on an individual level where each licensed fisherman is allowed 40 pots and recreational fishermen 14. However, it is important to notice that already during the 17^th^ century, the Dutch were reluctant to buy small lobster from Swedish suppliers and thus a functional MLS was already in practice. However, through the time series, the changes in the regulations have been minimal and therefore we can assume that they had a negligible effect on the trends observed here.

Using periodicity analysis, trend analysis and variability analysis we show how the population of European lobster in Sweden has changed from being regulated by density dependent population into an overexploited stock mainly regulated by fishery and climatic factors. Through undue exploitation, we have lost not only a valuable resource but most importantly an intriguing aspect of the dynamics of a natural population.

## Methods

### Database

Two different sources of historical data have been collated. The Swedish Rural Economy and Agricultural Societies (SREAS) collected data on number of fishermen, number of pots and total landings of lobster from 1875 to 1956. The data on number of pots, fishermen and lobsters landed were derived from 9 different areas, from Tistlarna, south of Göteborg, to Strömstad in the northern part of the Swedish west coast (Figure 1, area 10 omitted due to very small, or no, lobster catches). Data for 1893–94 and 1913–1918 were missing from the historical documents.

The second source of data is from a number of lobster fishermen that have provided us with Voluntary Catch Diaries (VCD). From 1938 to 2010, we obtained detailed VCD data with detailed information on catch and effort per pot from 33 fishermen along the Swedish west coast. Date of fishing was given in the VCD and transformed to Day of Year (DY, 1–365) to be included in analyses. Number of days at sea (DAS) was also given in the VCD. The effective soak time of a pot (S_t_) decreases as it is left at sea for several days. Effective soak time in relation to DAS (standardized to one day of fishing) follow the exponentially decaying relationship:
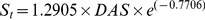
(1)


Parameters were fitted to a large data set which comprises mark and recapture data on lobster individuals in a no-take zone on the west coast of Sweden since 1992 (M.Ulmestrand pers. comm.). Approximately 5000 pots have been pulled over the years. Fitting was performed by least squares regression.

Gear efficiency has developed through the time series. During the1970′s, pots with an extra chamber were introduced. Those pots keep the bait longer, attracting lobster for a longer time and thus fish more efficiently, than pots without an extra chamber. Therefore, an experimental fishing was conducted to determine the relative catchability (*q*) of European lobster in the two different types of pots. The experiment revealed that the pots with an extra chamber fished on average twice as much as pots without it (*q* = 2.03±SE, p = 0.0012, F = 11.3, df = 76, described in details in Supporting Information S1). Thus, we used the catchability conversion factor, *q*, estimated from the experimental fishing to standardize CPUE, assuming a linear increase of the use of pots with an extra chamber from 1970 until 1980, when all pots where progressively mounted with an extra chamber (M. Ulmestrand, pers. comm.). Thus, standardized CPUE_VCD_ was calculated as:
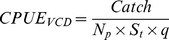
(2)where Np denotes number of pots.

#### Temperature

Physiological rates and behavior, such as growth and movement, are typically temperature dependent in European lobster [15], [16]. In order to assess the effect of temperature on the reconstructed dynamics of the population, two sets of temperature time-series were compiled using modeled data from surface water temperature in the Skagerrak (see details in Supporting Information S1). We calculated the average SST between June and September (SST_SUM_), to account for the effect that summer temperature may have on the recruitment to the fishery 3 to 5 years later, via individual growth and reproductive success. Moreover, we calculated the average temperature during September and October (SST_AUT_), when most of the catch occurs, to account for potential temperature-dependent variations in the catchability.

### Statistical analyses

#### Autocorrelation function – ACF

The standard tool to assess periodic fluctuations in time series is through the calculation of the autocorrelation function (ACF) of the corresponding time series [17]. This function measures the correlation of the time series, with a successively changing lag. At lag 0, the time series is perfectly correlated to itself (correlation coefficient *z* = 1). Changing the lag reveals alternate correlations in the time series. The statistical significance of each lagged correlation is given by the Bartlett bands (2/√n). The ACF can be further analyzed by constructing the partial ACF (PACF), which reveals the dominant lags within the time series, independently from the other lags in the ACF. In order to calculate the ACF, the time series needs to represent a stationary process, i.e., without temporal trends in mean or variance. Thus, we filtered the SREAS time series by linear detrending, removing the decreasing trend in the time series. The VCD data was strongly non-stationary and a linear detrending was not sufficient to remove the trends in mean and variance. The ACF and PACF of the VCD data were instead constructed on the residuals after fitting a generalized additive model to the data (see section below).

#### Generalized Additive Models

Models were fitted in order to standardize the effect of year, area, fishing day, lagged summer temperature, autumn temperature and CPUE lagged one year on the CPUE, and describe the main changes in the spatial distribution of lobster catches over time, generalized additive models (i.e. GAMs; [18]) were fitted to CPUE. Here we used a quasi-Poisson distribution with variance proportional to the mean and a log-link function in order to constrain the estimates to be positive. The quasi-likelihood approach assumes that the scale parameter *Φ* of the distribution is unknown, which makes it more suitable for over dispersed data than the classical Poisson distribution [19]. The full model was formulated as follows:
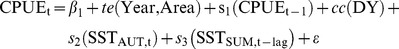
(3)where *β* is an overall intercept, *s* is an isotropic smoothing function (thin plate regression spline), *te* is a tensor product smoothing function, cc specifies a cyclic cubic regression spline, i.e., a penalized cubic regression spline whose ends match, and ε is an error term. The interaction term between year and area was included to investigate temporal changes in the CPUE from different areas along the Swedish west coast. Full and reduced models were compared based on both statistical significance and generalized cross validation (GCV; [19]). We further used ACF/PACF (above) to verify that the fitted GAM models returned residuals without autocorrelation. The GCV is a proxy for the models out-of-sample predictive mean squared error that includes a penalty for the number of parameters in the model. Therefore, a model with lower GCV has more explanatory power, and hence is preferred, compared to a model with higher GCV.

Smoothers on SST and Area were constrained to 4 knots to force the shape of the two variables to follow a positive or negative kurtosis, and to minimize the GCV-scores. The smoothers on DY were constrained to 6 knots. DY was not available for the SREAS-data set.

The GAMs on SREAS data where run by area for areas 8 and 9 to make sure that the AR(1)-patterns were found also on area level. We also ran modified GAMs on the VCD data using data for area 8 and 9 in two separate models with DY and Year as interaction terms to closely compare the SREAS and VCD-data (Supporting Information S1). We ran a model without DY as a predictor, restricting the data to include only the three first months of the season (when most of the catch is caught), to look for changes in CPUE without a seasonal effect.

#### Wavelet analysis

ACF is a powerful tool in visualizing fluctuations in populations. However, a prerequisite for this analysis is the stationary nature of the time series, i.e., the statistical properties, such as mean and variance, do not change over time. In modern fisheries stock development is often paired with a, usually negative, change in stock abundance [20]. In this particular example we were motivated to look for changes in the statistical characteristics of the time series, for example the change in periodicity through the time series. This violated the assumption of a stationary time series and compromised the use of the ACF. Wavelet analysis can cope with non-stationary time series and may also treat explicitly the temporal change in parameters through a local time-scale decomposition of the signal [21], [22] thus estimating the spectral characteristics as a function of time. We performed the wavelet analysis on both our time series to quantify the progressive change of periodicity through the time series.

Quantifying the variability in the VCD data set was given particular focus (Supporting Information S1). Variability in the untransformed CPUE VCD data was measured as Coefficient of Variation. This variability measure is defined as the standard deviation divided by the mean of the sample. The observed negative trend in stock size motivates the scaling of variability to the mean stock level. Variability measures are described in the Supporting Information S1. The wavelet analysis was performed in MATLAB, all other analyses were performed using R software (www.r-project.org).

## Results

**Figure 2:**
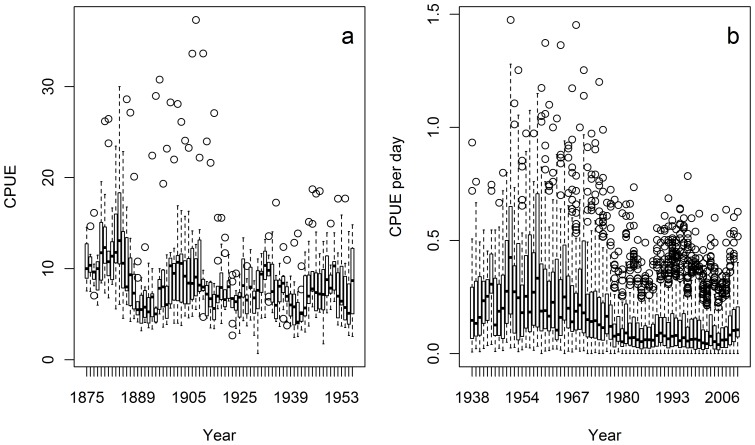
Two different time-series of catch per unit effort (CPUE) were collated in the current study, SREAS data (a) and VCD data (b). The SREAS data was aggregated by year making the scale CPUE per pot per year, distinguishing it from the VCD data where we could extract catches per pulled pot and day. The scale in (b) is CPUE per pot per pull.

**Figure 3:**
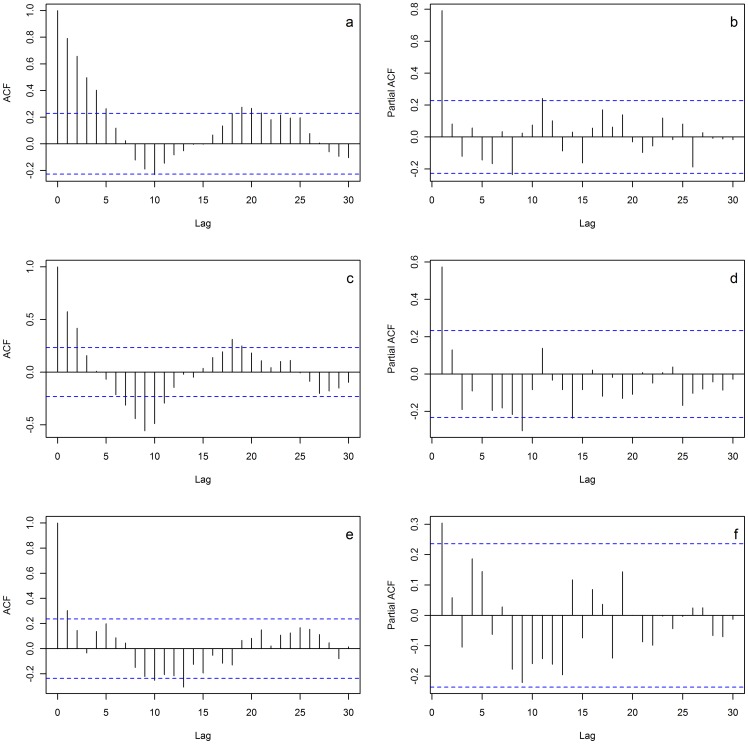
Autocorrelation, and partial autocorrelation, functions were used to analyze temporal lags in the time-series. We calculated ACF and PACF for linearly de-trended aggregated SREAS CPUE data (a, b), residuals of model H3 (c, d) and residuals of model L1 (e, f). Dashed lines are Bartlett bands showing approximate 95% confidence limits. For the SREAS data ACF and PACF was performed both on linearly de-trended CPUE values and the residuals of GAM model H3. In the case of VCD, showing non-stationary structuring, the ACF and PACF were performed only on the residuals of the GAM model L1.

**Figure 4:**
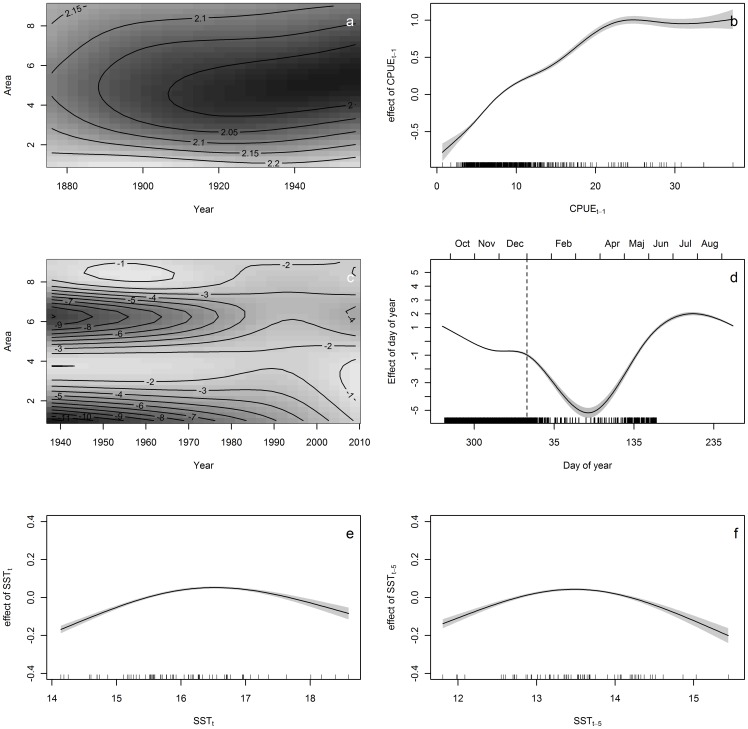
Several different GAMs were fitted to each of the data sets and best models were chosen by the GCV scores. Model effects of the best model of SREAS data (**a–b** H7, CPUE ∼ te(Year,Area) + s(CPUE_t–1_)+ ε) and VCD data (**c–f**, L1, CPUE∼ te(Year,Area) + s(DY) + s(SST) + s(SST5) + ε). **a**) shows the effect of the interaction term on CPUE and **b)** the effect on CPUE of CPUE lagged one year. **C–f** shows model effects of best model on VCD data (L1). Effects on CPUE from **c**) the interaction term, **d**) fishing day, **e**) lagged summer temperature SSTX and **f**) temperatures during fishing, SST_AUT_.

The collated data of the two sets of data are summarized in Figure 1 and 2. The first part of the times series (Figure 2a) revealed a strong autocorrelation structure and a significant lag of 1–3 years and a returning significant lag of up to 20 years (Figure 3a). The complementary partial autocorrelation function (PACF) suggested that the main structuring of this time-series is an autoregressive process (AR) of lag 1 (Figure 3 b). A number of GAMs were fitted to the SREAS data (H1–H7, Table S1). Models H1–H3 had similar fits. They differed only in the lag of SST_SUM_, and the 3, 4 or 5 year lag of average sea surface temperatures during summer only made a small difference to the GCV score, H3 had the lowest GCV (Table S1). The interaction component of Year and Area was significant as was the component average sea surface temperature during autumn (SST_AUT_). The effect of the interaction of Area over time (Year) showed lower CPUE in the central Bohuslän areas (Area 4–6) compared to the northern and southern areas. This effect became more pronounced over time through the SREAS data. However, models H1–H5 all showed autocorrelated residuals (as shown for H3 in Figure 3 c and d). When CPUE with one year lag was added as a predictor to the model H1 (as suggested by the PACF) all temperature components became insignificant. The ACF of the residuals of the model showed no autocorrelation. The reduced model H7 was thus chosen to be the best model (Table S1, Figure 4a–b). Supplementary models on the SREAS data per area returned autocorrelated residuals, unless a CPUEt-1 lag was introduced as a predictor (Supporting Information S1, Table S2).

**Figure 5:**
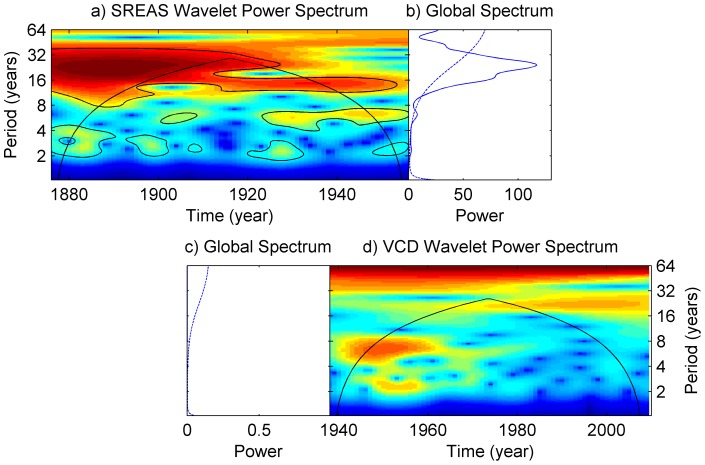
Wavelet analyses of periodicity of the two sets of lobster data. **a**) displays the wavelet power spectrum of the SREAS data where the dominant periodicity of ∼20 years becomes less pronounced as well as shorter through the time series. The color symbolizes the strength of the periodicity. Blue is weak, red is strong and contours indicate statistically significant periodicities. The cone of influence is drawn and suggests that any periods above are doubtful due to time series length. **b**) displays the global wavelet spectrum and the dotted line shows the corresponding confidence interval indicating the significance of the periodicities. **c**) and **d**) are the global power spectrum and the wavelet power spectrum of the VCD data respectively, showing no significant periodicity.

The wavelet analyses showed a change in periodicity through the time series. In the SREAS data, the wavelet analysis revealed a significant periodicity of ∼20 years (Figure 5a). Periodicity of 2–8 years was also identified. From the 1910′s the 20 year periodicity became weaker and the period shorter, and towards the end of the SREAS time series the periodicity of less than 8 years became more pronounced. The cone of influence makes the comparison of 20 year and 8 year periodicity unfeasible in the end of the time series (Figure 5a).

Several different GAMs were fitted to the VCD data (L1–L7, Table S1). Model L1 was chosen for its fit in terms of the deviance explained and the GCV score (Table S1, Figure 4c–f). A weak autocorrelation structure was observed in the residuals of L1 (Figure 3e and f). However, lagged CPUE was rejected because there was no relevant model improvement (L6, Table S1). In the supplementary models on the VCD-data the interaction between DY and Year in the reduced GAMs was significant. When DY was dropped from the models, explained variance also dropped and DY was kept in the best model (Supporting Information S1, Table S2).

The reduction in CPUE was evident during the 1950′s and 1960′s (Figure 2b) and thus we can exclude that this was simply an effect of the more efficient gear introduced in the 1970′s. The effect of DY has a typically seasonal pattern. Catches are largest at the opening of the fishing season (i.e. late September, DY ∼260) and declines towards the end of November (Figure 4d). The effect of temporally lagged averaged summer SST showed a dome shape relationship, with an optimum at approximately 13–14 C°. No significant periodicity was detected in the VCD data (Figure 5b). However, the tendency of periodic fluctuations portrayed by the wavelet analysis at 1940–1960 was of 2–8 years. These oscillations resemble those of the late SREAS data. They decay in the VCD time-series and do not return throughout the rest of the data (Figure 5b).

The number of outliers increases from the 1970′s and onwards (Figure 2b) and the variance in CPUE increases over the time series (n  =  70, p<<0.001, R^2^ = 0.31; see Supporting Information S1).

## Discussion

Cushing [23] stated that “Studies of observations in time series are used for two purposes. First, they reveal the variability of the numbers of populations [...]. The second aim is to study the extent to which the stabilization mechanism can damp or rectify the environmental variation. There is, of course, no real distinction between the two purposes because they are different facets of the single process by which recruitment is generated and populations are stabilized.” The crucial point here is that populations are not stabilized by the same process by which recruitment is generated. As Anderson et al. [5] have recently shown truncation of the population structure may affect intrinsic rates, such that recruitment is generated, not in relation to adult biomass, but rather inversely to adult biomass and magnified by environmental variability. On the other hand, a non-truncated population structure may have a stabilizing effect on population fluctuations. Nowadays there is a clear distinction between the two purposes of the time-series analysis, contrary to what Cushing claimed in 1975, and the key question is whether the signal of recruitment may be deciphered by ecological interactions or response to climate variability of the harvested stock.

The stabilization mechanisms mentioned by Cushing [23] do not modulate the environmental variability. The modulation of environmental cues happens through the filter of population size structure and the amplitude is due to the stochastic effects of individual encounters, for reproductive or other purposes, determining the outcome of a reproductive season. Recruitment, irrespective of the age or size composition of the recruiting class, should be treated as a demographic consequence of reproduction, with or without a density dependent transformation into recruits, and with or without environmental variability affecting the number of recruits. However, the regulation of the population abundance is a combination of the recruitment process and the density dependent response of the adult population to the recruitment pulses. We used statistical time-series models to disentangle the different regulatory mechanisms, which could generate the observed patterns in the available lobster data. Efforts to combine environmental and endogenous regulation in population development have been made throughout the history of ecology with recent additions to the enigma of the lemmings [24]. Rarely oscillations have been reported as endogenous.

Periodic oscillations may be triggered by introducing exploitation, dampened by increased exploitation and a very high level of exploitation may increase variability (fluctuations) in exploited stocks [4], [5], [6], [8]. These fluctuations may be changed to a shorter frequency as a result of truncated population structures and elevated adult mortality due to environmental variability [25]. The recent fluctuations of the lobster stock have not responded accordingly. Many aspects of the lobster biology is poorly known, partly due to the limited information on reproduction and the several year lag between hatching eggs and individuals recruiting into the fishery. Causes of the low variability between years for lobster may lie in the fact that individuals are 3–11 years old as they recruit into the fisheries. That is, several year-classes make up the recruiting class thus lagging environmental cues. This is parallel to the stabilization mechanism of Cushing [23] and the aggregated year-classes of juveniles will buffer for environmental variability not promoting it and also buffering the effects of environmental stochasticity. Obtaining data from the fishery, e.g. size and catch-at-age data, would allow us to individuate the processes shaping the observed dynamics.

A decline in stock size is often associated with intense and prolonged harvesting [20]. Intensive size selective harvesting leads to the truncation of the age structure, with larger and older individuals becoming rarer in the population. The combined effect of stock decline and age truncation in exploited fish populations has been proven to strongly influence variability in stock size [4],[5],[8]. Truncated populations show stronger fluctuations, as they tend to trace more closely stochastic environmental signals and increase growth rates. Increasing fluctuations have adverse effects on fish stocks [5] and might be a signal of approaching dynamic thresholds [26], beside the fact that they negatively affect fisheries industry decreasing the stability of the catches [5]. On the other hand, internal processes and species interactions were not found to produce periodic oscillations in most of the studied populations, which instead showed equilibrium dynamics [4], [27], [28] with fluctuations from other sources.

Our results show for the first time that exploitation removed the natural dynamics from the population fluctuations of European lobster, an otherwise inherent property of long-lived organisms with overlapping generations and lagged recruitment [29]. The oscillations are clearly visible in the data (Figure 1 and 2a). The period of the oscillation estimated from the ACF was 19–20 years with significant negative lags at 7–12 years, agreeing very well with the periodicity in the wavelet analysis (Figure 5a). The periodicity decays after 1930 and by the 1940 it is very weak, and it is not observed in the VCD data (Figure 5b). The population regulation is likely to be strongly dependent on endogenous causes as the ACF decays at larger lags [30]. If the cycle was generated by an exogenous factor, then the amplitude or height of the ACF should remain roughly constant as the lag gets larger, while if it decays with increasing lag, as in our data, then the causal process is likely endogenous [30]. Lobster is a long-lived species [31] and this implies that strong lagged effects on population dynamics might exist, occurring through competition for limiting resources and/or recruitment fluctuations. Unfortunately, as catch-at-age data are not available, we are unable to identify the process causing the observed periodicity. However, we argue that the observed strongly significant 20 year lag in the historical data series of lobster CPUE is likely to be an effect of density dependent regulation of both survival and reproduction in relation to the long life span of individuals.

The pronounced shift in the regulation of the population dynamics of the Swedish European lobster that we identified came about during the 1920 s and early 1930 s. This shift coincided with an increase in fishing effort of about 20% ([13]; this study]). During WWII, the shortage of fuel made fishermen predominantly reliant on rowing or sailing to pull their pots. Although effort and landings decreased during WWII, it was far from zero. The fact the fishery was sustained also during the war can be explained by the coastal nature of this fishery. European Lobster in Sweden is caught predominantly at 10–30 meters depth on rocky substrates. A lot of the shore is protected by a narrow archipelago of small islands making the lobster fishing grounds available even to small boats either sailed or rowed. Nevertheless, the stock increased during WWII likely due to reduced exploitation as showed for other species in the same area and period (i.e. [32], [33]). When peace was negotiated and the international trade resumed, the fishery went back to former levels and landings and CPUE increased. However, just after the end of WWII, the large increase in the intensity of the fishery quickly depleted the stock [this study]. In a few years, several age classes were fished out and the population was left at a low stock level. Only after several management efforts made during the 1980′s and the 1990′s the stock started to increase again, albeit slightly.

The pronounced oscillation in the SREAS data with a long periodicity was determined by an autoregressive process of first order – AR(1). The European lobster is a slow-growing stationary organism, which is quite difficult to lure into baited gear. Thus, strong autocorrelation in the lobster CPUE is to be expected in a naturally regulated system. The VCD data had a much less pronounced temporal structure (Figure 4e). There was a weak signal of density dependent regulation, but the use of an AR(1) term, although significant, did not notably improve the model (Table S1). Although not significant, the 2–8 year oscillations that are visible during 1940–60 in the wavelet analysis of the VCD data (Figure 5a), correspond to those oscillations of the same periodicity during the same time frame in the SREAS data (Figure 5c).

This study was based on catch data covering 1875–1956, when the SREAS collected catch and effort data per fishing area along the Swedish west coast. After 1956 there is no fishery-independent data available to support the results of this study and we have been forced to rely on a second set of data collected from diaries, VCD. The first set has good spatial and temporal coverage but no resolution on the individual fishermen. The VCD data has poorer spatial coverage, covers only a small fraction of the total fishery but has high resolution on individual fishermen and their catches through the season. The congruence of SREAS and VCD data is good (R2 = 0.19–0.50, Figure S3 in Supporting Information S1), and our conclusions hold also if we restrict the analyses to Areas 8 and 9 where most of the recent data was gathered. The temporal and spatial overlap strongly indicated that the two data sources tell a joint story, strengthening the patterns revealed by the analyses in this paper.

However, it is also important to stress that the mechanism behind such dynamics may be several and not easy to individuate by the GAM analyses. For example, resource limitation may cause metabolic retardation, slower growth and lower reproductive output and consequently a decreased recruitment. Also, shortage of shelters may cause larger natural mortality. These factors are also potentially density dependent and may cause periodic fluctuations in abundance. Another source of density dependent regulation is the highly variable stock-recruitment relationship [34]. Modeling studies of decapods have previously shown periodic or damped oscillations, primarily caused by overcompensating density dependence in stock-recruitment relationship (i.e. Ricker model of stock-recruitment relationship), but triggered by variable harvesting intensity [35]. However, an asymptotic formulation (i.e. Beverton & Holt model of stock-recruitment relationship) seems to be more realistic for lobster [34] and will not cause as dramatic fluctuations. We wish to stress that periodic, or damped, oscillations are most likely to occur at intermediate fishing intensities [6]. Overexploitation will push stock size to levels where overcompensation will not act on recruitment or mortality rates. This is the mechanism by which environmental variability strongly enters several fish time series [4], [5], [8]. The low level of variability between years in the lobster dataset during 1970–2010 indicates other mechanisms may be more important. One such mechanism may be a limitation in the finding of suitable mates in the population [36] and this Allee effect will hinder efficient reproduction and retard recovery of the stock.

We have insufficient data to verify an age, or size, truncation of the population, a common feature of exploited populations. Although the fishery is strongly size selective by the implementation of a minimum landing size, its strongest effect may be on the reduction of the total number of lobsters in the population rather than in the truncation of the population structure. However, the within year variability has increased substantially over the past 40 years (Figure S1b in Supporting Information S1). This is likely caused by the observed decline in the density of the population. On the other hand, variability between years is rather small (Figure S1a in Supporting Information S1) contrary to the predictions of other authors (e.g. [4], [5], [8]). This can be explained by the fact that European lobster is a long lived species with a relatively low fecundity compared to other decapods, and it is difficult to lure into pots. Daily catches have become more random, due to stochastic effects in a small size stock. Thus, fewer pots are visited by lobsters when population density decreases. Moreover, in a small population the geographic distribution will also become patchier. This will result in lower average catch, with more zeros and with few random events of large catches, causing higher within year variability as the stock declines.

As for many other species (i.e. [37]), the shifting baseline syndrome [38] has altered our perception of the lobster stock. The anecdotes of pots full of lobsters in the archipelago, awaiting export to England [39], appear nowadays as dreams passed on by the older generations. However, in the case of European lobster, the shifting baseline syndrome has not only shifted our perception of the state of the stock, but also its dynamics. The diary data from 1938 until today is limited, in space as well as in the relation to the fishing community, covering only a couple of percent of the total effort in the fishery. Better data coverage would produce more precise estimates of the total catches and effort as well as provide catch-at-age and/or size. If we could estimate F (from size distributions) we could also establish a management system based on an F target similar to that of the American lobster in the Gulf of Maine [40]. To implement this type of adaptive management strategy in Sweden needs an extension of the current data sampling.

It is important to point out that from the 1950 s and onwards there are no comprehensive statistics on the total yearly catch of European lobster in Sweden. The fishery is today dominated by recreational fishermen who do not need to report or declare their catches. Better information on the total effort and landings would greatly simplify a formulation of an adaptive management regime. Today, management actions of lobster fishery regulations have to rely on the catches of a few professional fishermen and their detailed journals (the VCD data in this study) and assumptions on reproductive biology, of which fairly little is known [34], [41]. Studies like ours, revealing changes in the regulation of dynamics caused by an inadequately regulated fishery, directs attention to the implementation of a sound data collection. To satisfactorily evaluate management actions we need useful measures on total effort and catch. With such data collection in place we could reach an adaptive management for the Swedish lobster.

In the terrestrial systems, periodic fluctuations, which may be caused by density dependent regulation [2] and species interactions [42], [43], have been extensively described [3]. In marine systems, examples of corresponding dynamics are rare (i.e., [44]), and endogenous processes have previously been shown to be of minor importance in generating fluctuations in harvested fish populations [4]. Turchin [3] claimed that oscillatory systems are potentially easier to predict compared to a stable but noisy system. However, an oscillatory system is not qualitatively different from a stable one, with the apparent dissimilarity linked to quantitative differences in the values of the parameters [3]. The case depicted here is a classic example of overexploitation, where the overexploitation has not simply reduced the abundance of the stock but it has also eradicated the natural harmonic oscillatory behavior of the population dynamics.

## Supporting Information

Supporting Information S1 **Supporting information.**
(DOCX)

Table S1 GAM models fitted to the full SREAS and VCD data.
(DOCX)

Table S2 GAM models fitted to partial or aggregated data sets.
(DOCX)

Table S3 ANOVA-table showing statistical differences in catch for different pots.
(DOCX)
