# Supplementary material for: Multi-Annual Fluctuations in Reconstructed Historical Time-Series of a European Lobster (Homarus gammarus) Population Disappear at Increased Exploitation Levels
Source: PLoS One. 2013 Apr 3;8(4):e58160. doi: 10.1371/journal.pone.0058160 (PMC3616055; doi:10.1371/journal.pone.0058160)
Supplement: Supporting Information S1 — Supporting information. (DOCX) [file pone.0058160.s001.docx]

**Supporting Information S1**

Database

Data from Area 1 was, from 1891, reported as subdivided into 1A, 1B and 1C. However, they were merged for the entire Area 1 in the analysis as detailed spatial information was not available during the entire time series. Area 10 (Idefjorden) contained very little catch for very few years and was omitted from the study. For the years 1893 and 1894, data was reported for the entire area and was not used. In 1914-1918 data contained only landings and no measure of effort and was therefore also not included in the analyses.

The official lobster landings can be traced back to 1875, and only since the 1970’s they are unreliable, due to the intensified recreational fishery taking a large part of the total landings. In Figure S2 in Supporting Information S1 we display the official landings (in tonnes) and the effort in the fishery, measured as thousands of pots, disregarding technical creep.

Temperature

Met Office Hadley Centre’s sea ice and sea surface temperature (SST) data set, HadISST1, was used to collate a time series of temperatures for the Skagerrak. This dataset is a combination of monthly globally complete fields of SST and sea ice concentration on a 1◦ latitude-longitude grid from 1870 (obtained from http://badc.nerc.ac.uk, see reference [2] for more details). We selected SSTs from the grid cells that covered the study area.

Surface temperatures may not be ideal for a demersal species like the European lobster. However, European lobsters are active around isoclines and thus are likely to be affected by surface temperatures. There is also a rather good correspondence between surface and bottom temperatures in the North Sea, except in the summer [3, 4]. Monthly averages of June-September were assumed to represent summer average temperature (SST_SUM_) and September-October temperatures were assumed to represent average autumn average temperature (SST_AUT_). Summer temperatures are assumed to directly affect growth of individuals and their reproductive success. This would result in a lagged effect of summer temperature on recruitment to the fishery between 3 to 5 years later. The autumn temperatures, on the other hand, potentially affect the fishery directly. The bulk of the catches occur in September and October, therefore the autumn temperature is assumed to affect the catchability of European lobsters without a lag. At low temperatures lobsters are inactive but at higher temperatures lobsters become quite mobile and will encounter pots more frequently. However, if temperatures reach 22 degrees Cº ammonia start to build up in lobster and individuals will try to find colder waters.

Temporal trend in the coefficient of variation

The numbers of outliers in Figure 2b increase for the 1970’s and onwards thus the data are better treated graphically on a log scale (Figure S1a in Supporting Information S1). The decrease in mean CPUE is apparent but the percentiles do not seem to change substantially. The measure of variability, the Coefficient of Variation (CV), is calculated as the standard deviation divided by the mean. Regressing CV against years revealed a strongly significant positive trend in the CPUE variability over time (Figure S1b in Supporting Information S1).

Efficiency of the gear

Pots have developed during the 400 years of harvesting in Scandinavia. Pots were originally made of wood but today plastic rings with nylon webbing or even metal frames with nylon webbing are widely used. It was not until the 1970’s that pots with an extra parlor became widespread in the fishery. A parlor pot is equipped with one (or more) extra parlor that is easily entered from the chamber, but it is difficult to escape. Moreover, in the parlor the caught individuals cannot reach the bait, and the pot is therefore attractive to lobster and thus operable for a longer time period. The parlor and the main chamber are both equipped with escape gaps of usually 54 mm diameter. Thus, we needed to evaluate the effect of gear development on catch per unit effort (CPUE) to be able to standardize the effort over time and its effect on the trend in the lobster catches.

To do so, an experiment was designed to analyze the difference in the mean catch between parlor pots and pots without parlors. The experiment was conducted in an area closed to a no-take zone, Kåvra, has n this small area (2.1 km^2^) surrounding the island of Kåvra, NW of Lysekil (N 19.7’, 11º 21.7’) and located in the middle of our study area (Area 7A in Figure 1), the density of lobster is much larger than in neighboring, harvested, areas. In this area catches are guaranteed to be high and individuals range in size as in an unexploited population. This is an ideal experimental lobster laboratory where fishing with different gear was easily tested.

The fishing vessel was cruising over feasible lobster habitat and when good rocky bottom was encountered a pot with a parlor was deployed and, separated by 5 meters of rope, a second pot, without a parlor, was deployed. Pots were left at sea for 1 to 4 days before to be pulled. Fishing was performed from 20^th^ of July to 7^th^ of August 2009, a period when lobsters in Sweden are very active and motile. A total of 81 pulled paired pots and a total catch of 102 lobsters where used for the analysis of the difference in catch efficiency between the pot types.

The data was analyzed using a two-way ANOVA with paired observations and with the type of pot as single factor. The test showed a significant difference between the two types of pots (Table S3). The parlor pots caught on average twice the amount of lobster than the pots without a parlor.

SREAS to VCD alignment

The SREAS data cover records of lobster fishery in 9 areas along the West Coast of Sweden from 1875-1956. The data include number of caught lobster, lumber of fishermen and number of pots in each area.

The VCD data was individual based on a fisherman scale including information of deployment times for pots, catch per pot and date of fishing. The bulk of the diary data (VCD) is from area 8-9 (17 of 33 diaries), where we also have a complete coverage in time. The later part of the data series has a wider spatial coverage, including areas 1, 4, 6 and 7 (Figure 1). From 1938 to 1956, the period of overlap the data covered 1-4 fishermen (all in areas 8 and 9) with extended licenses for 75 pots. This corresponds to a total of 75 – 300 pots.

The R^2^ of the correlation between the datasets (Figure S3 in Supporting Information S1) for areas 8 and 9 was 0.19, which we regard as good, considering the different structures of the data. The conversion from data on a daily basis to a yearly basis would cause some discrepancy because there is a strong seasonal component in the determining of catches (this work). Predicting VCD data from SREAS data through linear regression from 1951 through the last years of overlap (when data was collected from 300 pots) the R^2^ was 0.50 (n=7, p=0.08). Although above the commonly used p-value threshold of 0.05 we found that the two sets of data capture trends in fluctuations similarly (FigureS3 in Supporting Information S1). We consider this as a census fishery, for which stochasticity is usually higher when fewer fishermen were part of the diary data.

GAMs

Two secure that the transfer of dynamics from the SREAS to VCD data sets happen on a area scale we ran additional GAMs on the SREAS data set for areas 8 and 9 separately and showed that incorporation of a CPUEt-1 lag is essential to remove the autocorrelation in the data on a area level (H8lag and H9lag, Table S2, Figure S4A in Supporting Information S1). The ACF of the residuals (Figure S4B in Supporting Information S1) showed the lack of autocorrelated residuals of the areas 8 and 9 -models. The density dependent predictor CPUE_t-1_ was found to be essential in explaining the data, and causing the periodic oscillations in the SREAS data.

The strong effect of Day-of-Year (DY) on CPUE in the VCD data is a combined effect of lobster being taken out of the system through harvesting, and the dropping activity patterns due to decreased temperatures during winter. We ran a GAM on data from the first three months of the season to see if we could capture biomass trends over years without DY as a predictor (L_season_, Table S2). This model explained less than 30% of the data, compared to 50% or more for the models including DY (Table S1). We ran a modified GAM on data on area 8 and 9 in separate models using DY and Year as interaction terms to closely compare the SREAS and VCD-data. The interaction between DY and Year for the best model of area 8 and 9 was significant (L8 and L9, Table S2). The effect of DY on the CPUE in the VCD data thus changes through time. However, the general pattern of the best catches occurring during early season remained. When DY was dropped as a predictor from the model (L_season_, Table S2) and a partial dataset was used (the first three months of the season only) the explained variance also dropped. DY was considered our best way of incorporating the fishery explicitly in the modeling and also capturing other temporal trends. DY hence remained as a predictor in the best model. We concluded from this that the dynamics displayed by the time series are qualitatively comparable disregarding scale issues. The changes we described for the full datasets were also seen on a per area level.

**Cited literature**

1. Axelsson G (1944). Det Svenska hummerfisket och fångstens avsättning. Seminariearbete i ekonomisk geografi med råproduktlära vid Handelshögskolan i Göteborg hösten 1942. Göteborg: Wezäta-Göteborgslitografen AB.
2. Rayner NA, Brohan P, Parker DE, Folland CK et al. 2006: Improved analyses of changes and uncertainties in marine temperature measured in situ since the mid-nineteenth century: the HadSST2 dataset. J Climate 19:446-469.
3. Elliot AJ, Clark T, Li Z (1991) Monthly distributions of surface and bottom temperatures in the northwest European shelf seas. Cont Shelf Res 11:453-466.
4. Clark RA, Fox JC, Viner D, Livermore M (2003) North Sea cod and climate change – modelling the effects of temperature on population dynamics. Global Change Biol 9: 1669–1680. doi: 10.1046/j.1529-8817.2003.00685.x.


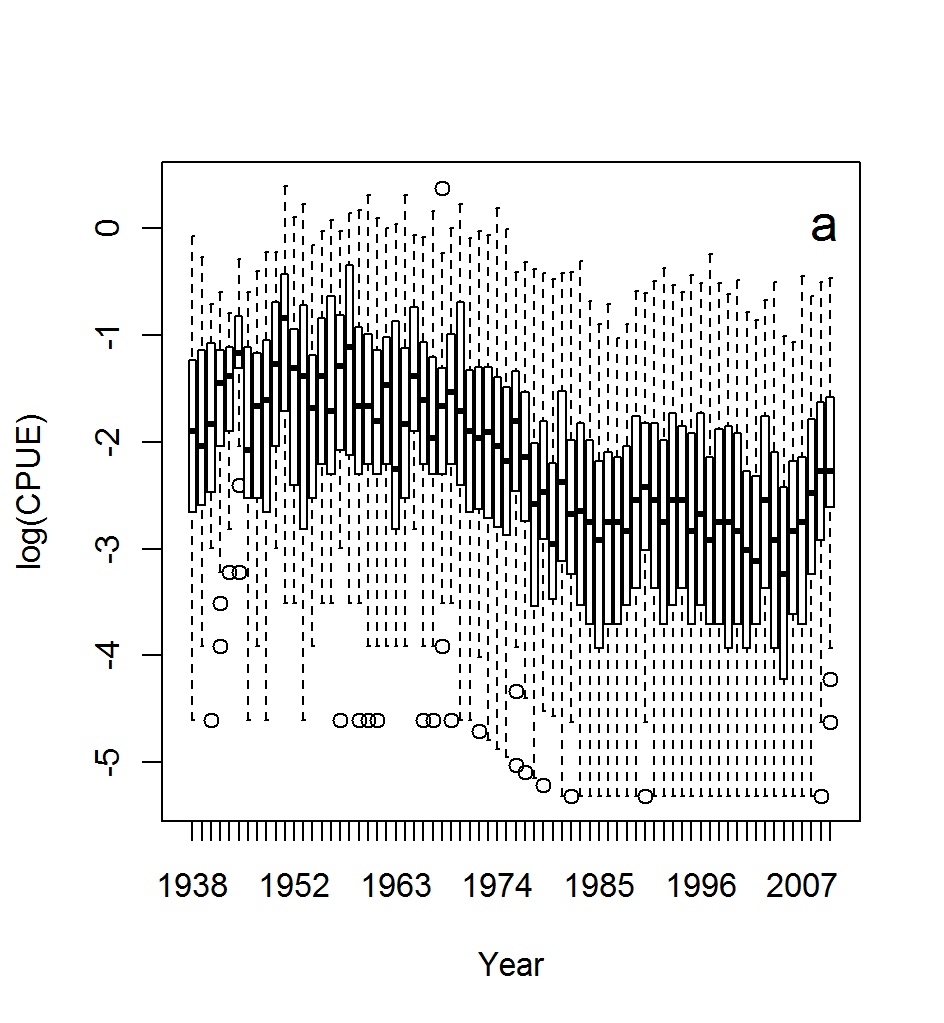
**
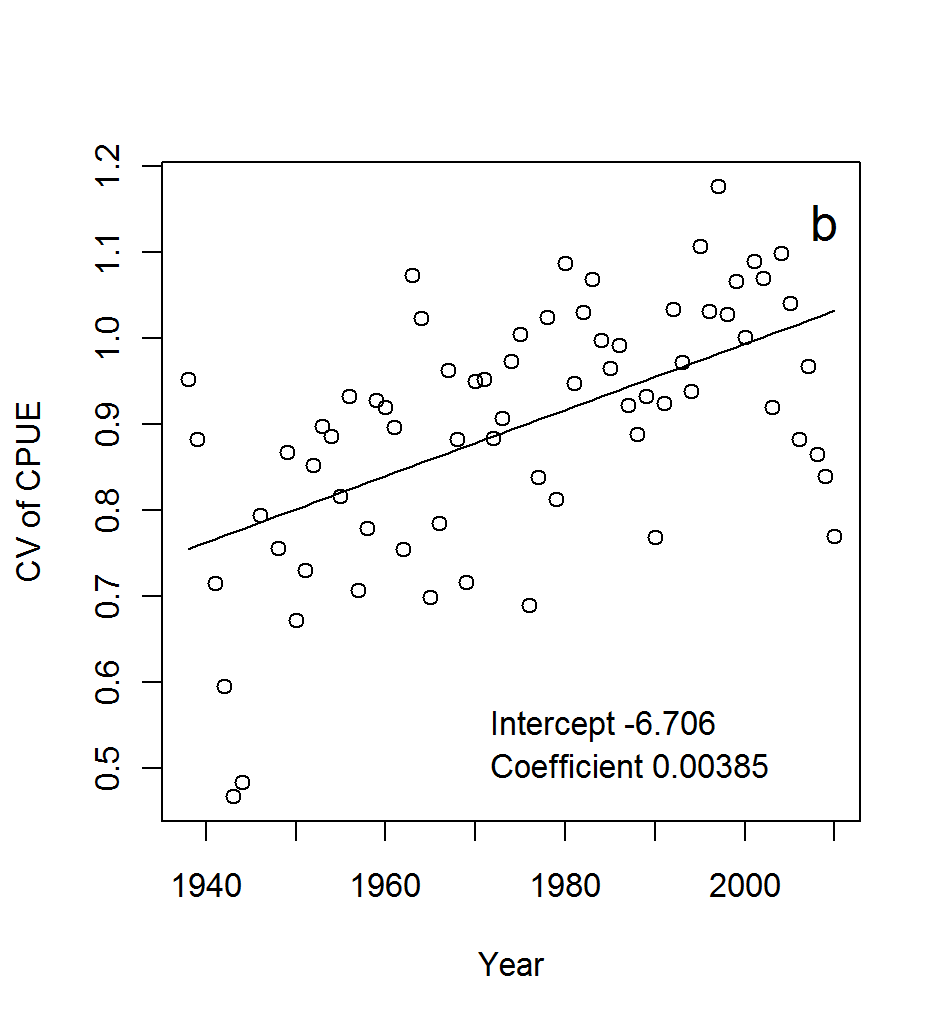
**

**Figure S1**. The CPUE VCD data on a log scale (**a**) indicating a stationary variance although a decreasing median CPUE. The temporal trend of the CV of CPUE (**b**) showed a positive trend over time (n=70, p<<0.001, R^2^ = 0.31).

**
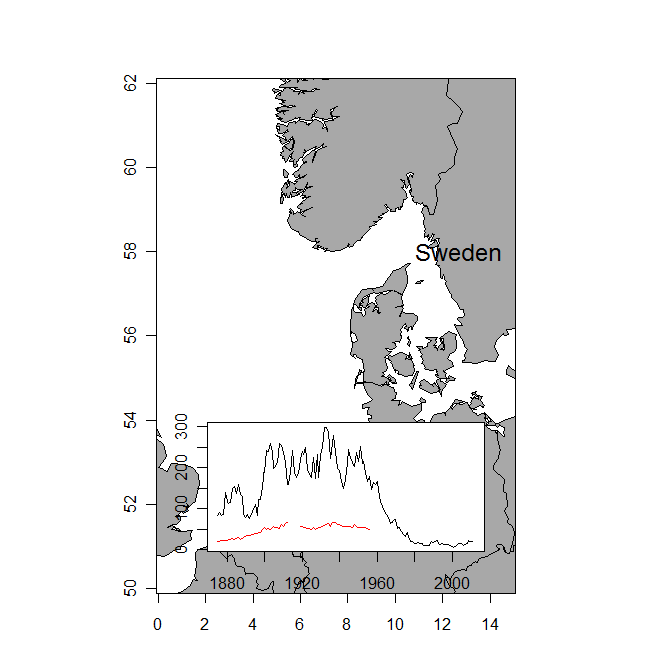
**

**Figure S2.** Map over the study area. The inner panel displays landings (black) and effort (red) in thousands of lobsters and pots respectively.


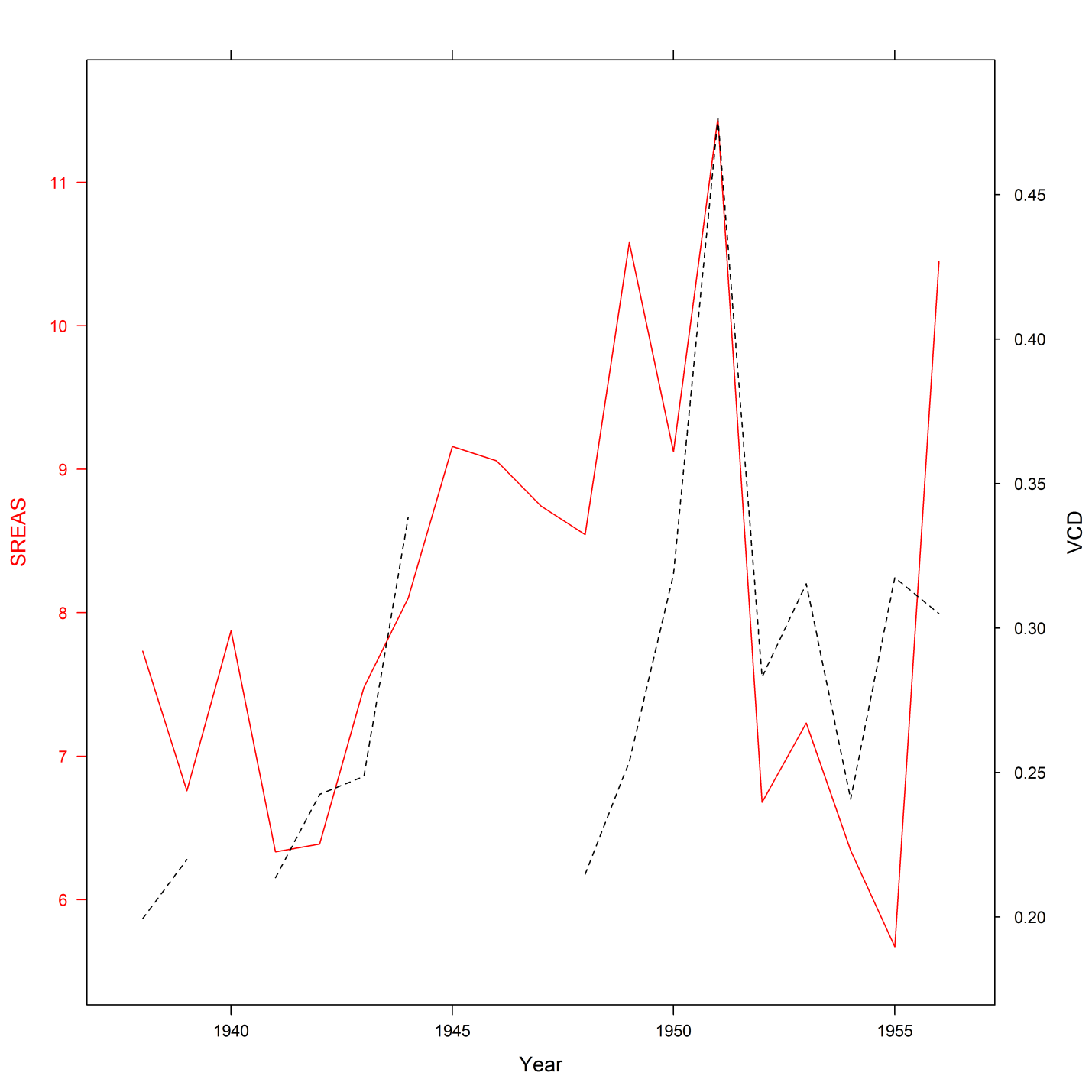


**Figure S3**. Alignment of VCD and SREAS data showing high degree of correlation through the last years of overlap.


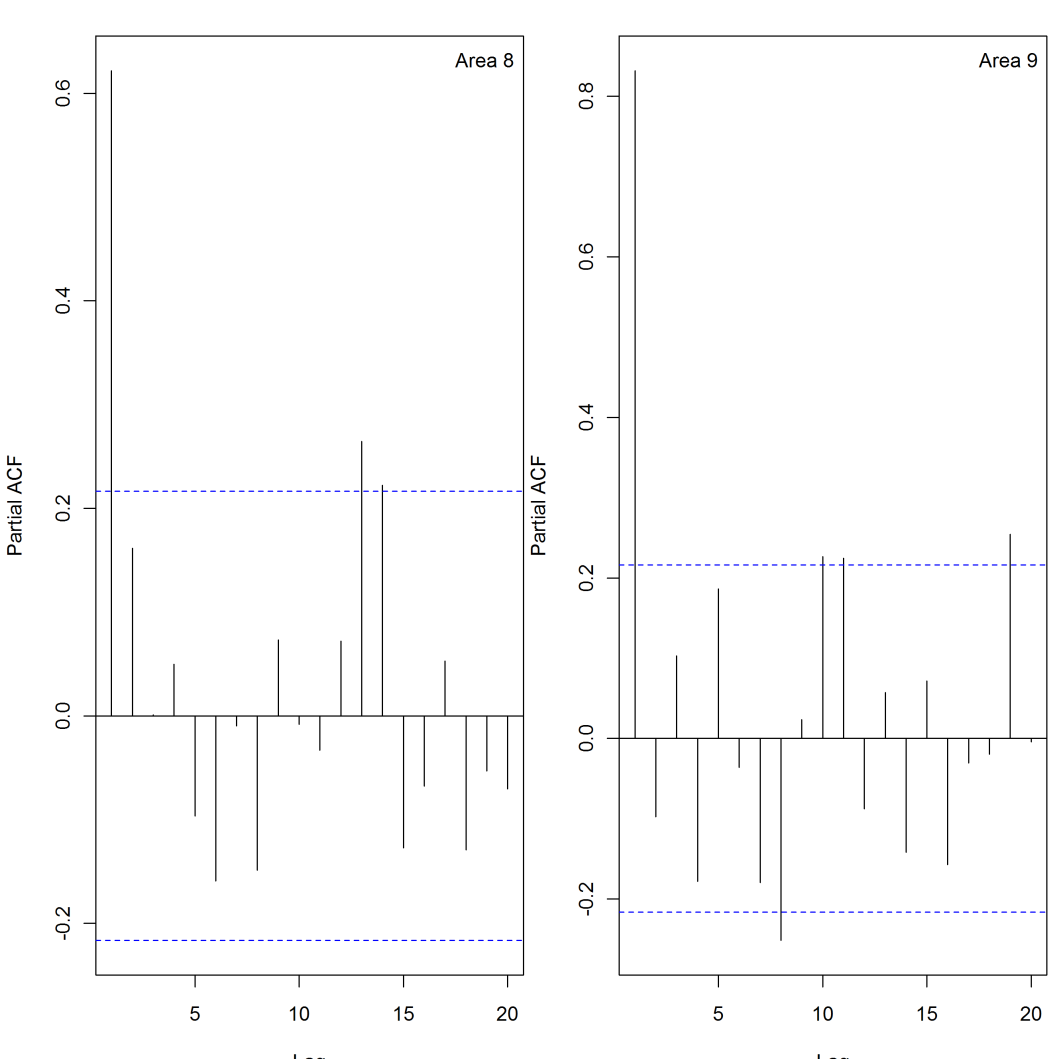


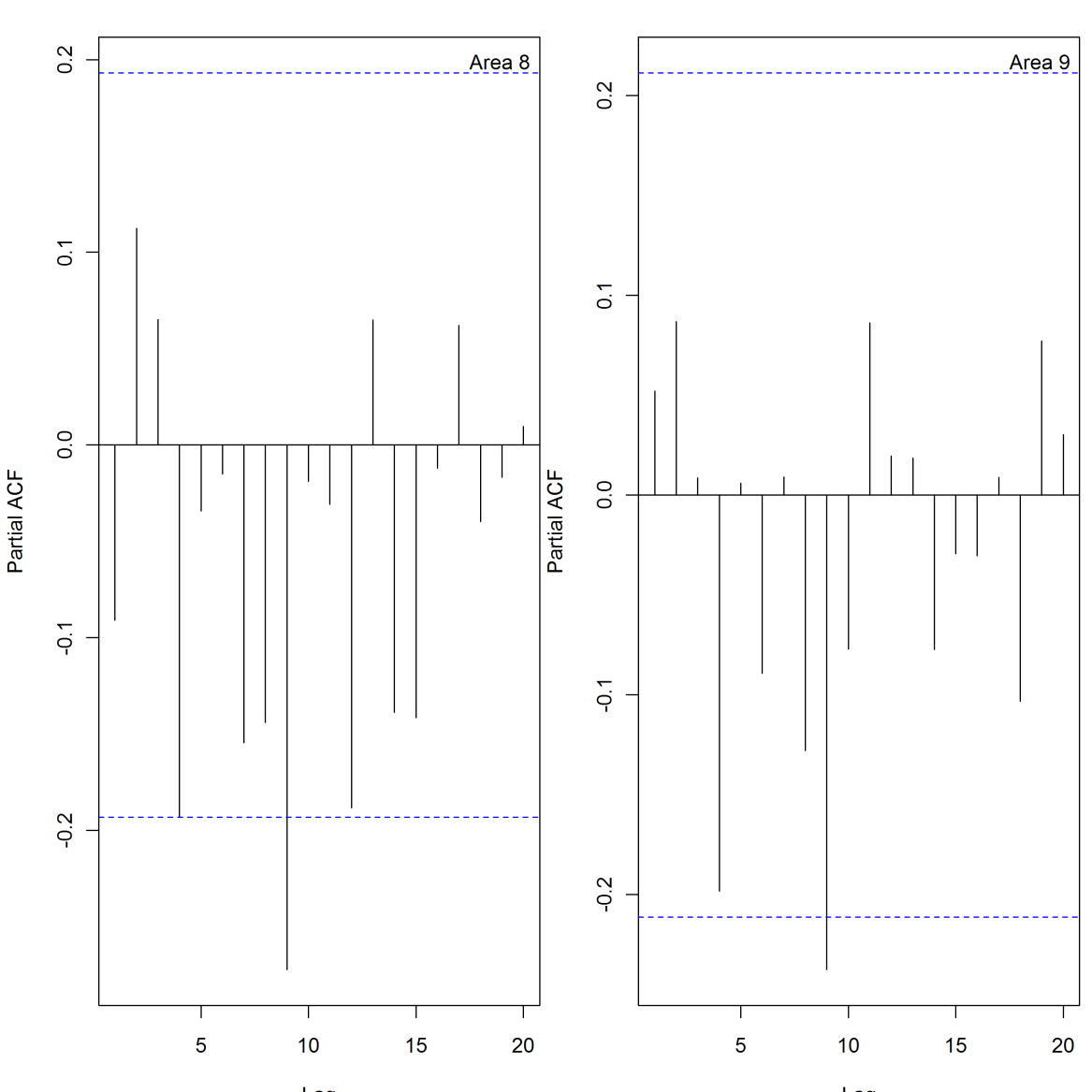


**Figure S4**. Partial autocorrelation show the dependence of the data on lags in the data. A) CPUE in the SREAS data from areas 8 and 9 are highly autocorrelated with a AR(1) process producing most of the autocorrelation. B) Partial autocorrelation of residuals (from GAM H8 and H9) show that the CPUEt-1 term in the GAM removed the autocorrelation found in the CPUE.
