# Supplementary material for: Multi-Annual Fluctuations in Reconstructed Historical Time-Series of a European Lobster (Homarus gammarus) Population Disappear at Increased Exploitation Levels
Source: PLoS One. 2013 Apr 3;8(4):e58160. doi: 10.1371/journal.pone.0058160 (PMC3616055; doi:10.1371/journal.pone.0058160)
Supplement: Table S1 — GAM models fitted to the full SREAS and VCD data. (DOCX) [file pone.0058160.s002.docx]

**Table S1.** Generalized additive models to explain CPUE of European lobster for the SREAS (1875-1956) and VCD (1938-2010) time series. Model fit is given as DEV% and General Cross Validation. SST3 - SST5 denote average Sea surface temperature during summer with a 3-5 year lag respectively. SST denotes average Sea surface temperature during main fishing season. Significance of descriptors is *** if nothing else is stated. Best model was chosen on the conditions of GCV, DEV %, and the significance of included terms.

| N | GAM fitted to CPUE_SREAS_ | DEV % | GCV | Sign. | Comment |
| --- | --- | --- | --- | --- | --- |
| H1 | te(Year,Area) + s(SST) + s(SST3) | 58.1 | 1.0302 | SST3 n.s., SST ** |  |
| H2 | te(Year,Area) + s(SST) + s(SST4) | 58.1 | 1.0302 | SST4 n.s., SST ** |  |
| H3 | te(Year,Area) + s(SST) + s(SST5) | 58.4 | 1.0252 | SST5 n.s., SST ** |  |
| H4 | te(Year,Area) + s(SST) | 58.1 | 1.0272 | SST ** |  |
| H5 | te(Year,Area) | 57 | 1.0465 |  |  |
| H6 | te(Year,Area) + s(CPUEt-1) + s(SST) + s(SST5) | 75.2 | 0.6059 | SST n.s., SST5 n.s. |  |
| **H7** | **te(Year,Area) + s(CPUEt-1)** | **74.4** | **0.6076** |  | **best model** |
| N | GAM fitted to CPUE_VCD_ |  |  |  | Comment |
| **L1** | **te(Year,Area) + s(DY) + s(SST) + s(SST5)** | **55** | **0.06** |  | **best model** |
| L2 | te(Year,Area) + s(DY) + s(SST) + s(SST5) | 50.7 | 0.0725 |  | Increased catchment during 70-80, q ranging 1 - 1.5 |
| L3 | te(Year,Area) + s(DY) + s(SST5) | 54.7 | 0.0604 |  |  |
| L4 | te(Year,Area) + s(DY) | 54.5 | 0.0607 |  |  |
| L5 | te(Year,Area) | 28.3 | 0.0955 |  |  |
| L6 | te(Year,Area) + s(DY) + s(SST) + s(SST3) + s(CPUEt-1) | 55.5 | 0.0592 |  |  |
| L7 | te(Year,Area) + s(CPUEt-1) | 28.5 | 0.095 |  |  |
