# Supplementary material for: Multi-Annual Fluctuations in Reconstructed Historical Time-Series of a European Lobster (Homarus gammarus) Population Disappear at Increased Exploitation Levels
Source: PLoS One. 2013 Apr 3;8(4):e58160. doi: 10.1371/journal.pone.0058160 (PMC3616055; doi:10.1371/journal.pone.0058160)
Supplement: Table S2 — GAM models fitted to partial or aggregated data sets. (DOCX) [file pone.0058160.s003.docx]

**Table S2.** Generalized additive models to explain CPUE of European lobster for the SREAS (1875-1956) time series tested on Areas 8 and 9 separately. Model fit is given as DEV% and General Cross Validation. The lagged CPUE-description was needed in order to get random residuals. Model N8 run with partial data and no season information.

| **no** | **GAM fitted to CPUE_SREAS_** | **DEV %** | **GCV** | **Sign** | **Comment** |
| --- | --- | --- | --- | --- | --- |
| H8 | s(Year) + s(SST) + s(SST5) | 59 | 0.2545 | Year***, SST and SST5 = ns | Autocorrelated residuals |
| H9 | s(Year) + s(SST) + s(SST5) | 72.5 | 0.9746 | Year***, SST and SST5 = ns | Autocorrelated residuals |
|  |  |  |  |  |  |
| H8 lag | s(Year) + s(CPUEt-1) | 51.3 | 0.251 | CPUEt-1***, Year = ns | No autocorrelated residuals |
| H9 lag | s(Year) + s(CPUEt-1) | 72.2 | 0.7468 | CPUEt-1***, Year = ns | No autocorrelated residuals |
|  | **GAM fitted to CPUE_VCD_** |  |  |  |  |
| Lseason | te(Year, Area) + s(sst1) + s(sst5) | 28.9 | 0.0947 | SST = ns | model run with catch data for first three months of season |
| L8 | te(Year, DY) + s(sst) + s(sst5) | 60.5 | 0.0603 | All sign |  |
| L9 | te(Year, DY) + s(sst) + s(sst5) | 51.4 | 0.0589 | All sign |  |
|  |  |  |  |  |  |
