# Supplementary material for: Multi-Annual Fluctuations in Reconstructed Historical Time-Series of a European Lobster (Homarus gammarus) Population Disappear at Increased Exploitation Levels
Source: PLoS One. 2013 Apr 3;8(4):e58160. doi: 10.1371/journal.pone.0058160 (PMC3616055; doi:10.1371/journal.pone.0058160)
Supplement: Table S3 — ANOVA-table showing statistical differences in catch for different pots. (DOCX) [file pone.0058160.s004.docx]

**Table S3**

Summary of ANOVA

| SUMMARY |  |  |  |  |  |  |
| --- | --- | --- | --- | --- | --- | --- |
| *Groups* | *Count* | *Sum* | *Average* | *Variance* |  |  |
| 2 | 39 | 24.91 | 0.63 | 0.25 |  |  |
| 0 | 39 | 12.25 | 0.31 | 0.11 |  |  |
|  |  |  |  |  |  |  |
|  |  |  |  |  |  |  |
| ANOVA |  |  |  |  |  |  |
| *Source of Variation* | *SS* | *df* | *MS* | *F* | *P-value* | *F crit* |
| Between Groups | 2.056 | 1 | 2.05 | 11.32 | 0.001 | 3.96 |
| Within Groups | 13.802 | 76 | 0.18 |  |  |  |
|  |  |  |  |  |  |  |
| Total | 15.859 | 77 |  |  |  |  |
